# Increased Screen Time Is Associated With Alcohol Desire and Sweetened Foods Consumption During the COVID-19 Pandemic

**DOI:** 10.3389/fnut.2021.630586

**Published:** 2021-03-24

**Authors:** William R. Tebar, Diego G. D. Christofaro, Tiego A. Diniz, Mara Cristina Lofrano-Prado, Joao Paulo Botero, Marilia de Almeida Correia, Gabriel G. Cucato, Raphael Mendes Ritti-Dias, Wagner Luiz do Prado

**Affiliations:** ^1^São Paulo State University (UNESP), Faculty of Science and Technology, Presidente Prudente, Brazil; ^2^University of São Paulo—USP, Post-graduation Program in Cell and Tissue Biology, São Paulo, Brazil; ^3^Independent Researcher, Mentone, CA, United States; ^4^Federal University of São Paulo—UNIFESP, Santos, Brazil; ^5^Universidade Nove de Julho—UNINOVE, São Paulo, Brazil; ^6^Department of Sport, Exercise and Rehabilitation, Northumbria University, Newcastle upon Tyne, United Kingdom; ^7^California State University San Bernardino, San Bernardino, CA, United States

**Keywords:** sedentary behavior, dietary pattern, substance use, COVID-19, pandemic

## Abstract

**Background:** Elevated screen time has been associated with addictive behaviors, such as alcohol and sugar intake and smoking. Considering the substantial increase in screen time caused by social isolation policies, this study aimed to analyze the association of increased screen time in different devices during the COVID-19 pandemic with consumption and increased desire of alcohol, smoking, and sweetened foods in adults.

**Methods:** A sample of 1,897 adults with a mean age of 37.9 (13.3) years was assessed by an online survey, being composed by 58% of women. Participants were asked whether screen time in television, cell phone, and computer increased during the pandemic, as well as how much time is spent in each device. Closed questions assessed the frequency of alcohol and sweetened food consumption, smoking, and an increased desire to drink and smoke during the pandemic. Educational level, age, sex, feeling of stress, anxiety, depression, and use of a screen device for physical activity were covariates. Binary logistic regression models considered adjustment for covariates and for mutual habits.

**Results:** Increased television time was associated with increased desire to drink (OR = 1.46, 95% CI: 1.12; 1.89) and increased sweetened food consumption (OR = 1.53, 95% CI: 1.18; 1.99), while an increase in computer use was negatively associated with consumption of alcohol (OR = 0.68, 95% CI: 0.53; 0.86) and sweetened foods (OR = 0.78, 95% CI: 0.62; 0.98). Increased cell phone time was associated with increased sweetened food consumption during the pandemic (OR = 1.78, 95% CI: 1.18; 2.67). Participants with increased time in the three devices were less likely to consume sweetened foods for ≥5 days per week (OR = 0.63, 95% CI: 0.39; 0.99) but were twice as likely to have sweetened food consumption increased during pandemic (OR = 2.04, 95% CI: 1.07; 3.88).

**Conclusion:** Increased screen time was differently associated with consumption and desire for alcohol and sweets according to screen devices. Increased time in television and cell phones need to be considered for further investigations of behavioral impairments caused by the pandemic.

## Introduction

The polices of social isolation to counteract the spread of the COVID-19 pandemic has caused an increase in screen time (1), which is associated with impairments in mental and general health (2).

The elevated time in screen devices has been associated with addictive behaviors since before the pandemic, including alcohol consumption (3), smoking (4), and sugar intake (5). In this sense, due to the substantial increase in screen time caused by social isolation, it is possible that people are being very exposed to unhealthy advertisements in television and social media (6–8), as well as excessive information about the pandemic (9), which has been associated with poor mental health status (10) and may lead to an increase in addictive behaviors, mainly in regard to alcohol and tobacco (11–13). Besides that, excessive sugar intake has also been considered as an addiction (14), since high-palatable foods activate brain regions, which are responsible for pleasure and reward, as drugs (15). Sugar intake from sweetened foods was prospectively associated with poor mental health (13).

To test this hypothesis, this study aimed to analyze the association of increased screen time in different devices during the pandemic with alcohol consumption and the desire to drink, smoking and the desire to smoke, and high-sweetened food consumption and its increase during the pandemic in adults.

## Methods

This electronic survey research was conducted in Brazil, with data collection between May 5 and May 17, 2020. Participants were invited through social media (Facebook, Twitter, Instagram, and WhatsApp) to answer an online questionnaire using the Google Forms platform (Mountain View, CA, USA). This study was approved by the Universidade Nove de Julho' Ethics Committee before data collection (CAAE #30890220.4.0000.5511). Inclusion criteria was to be ≥18 years old and answer all the questions. Participants did not identify themselves, and their answers were only included in the sample if they authorized it before the protocol started, after reading the Informed Consent Form. All procedures followed the national legislation and the Declaration of Helsinki.

The survey was composed by 70 questions divided into seven domains: (1) personal information; (2) COVID-19 personal care; (3) physical activity; (4) eating behavior; (5) health risk habits; (6) mental health; and (7) overall health (16). For the purpose of the present study, specific questions were considered from personal information (age, sex, and educational level), COVID-19 personal care (use of screen device for the practice of physical activity), eating behavior (weekly frequency of sweetened food consumption, and increased sweetened food consumption during the pandemic), health risk habits (screen time, alcohol consumption, desire to drink during pandemic, smoking, and desire to smoke during pandemic), and mental health (feeling of anxiety, stress, and depression).

### Screen Time

The daily hours spent in television viewing, cell phone, and computer were used to assess the screen time of the sample, through specific questions for each device: “How many hours per day do you spend on television/cellphone/computer during the COVID-19 pandemic?” Responses were: (i) <1 h/day; (ii) 1 h/day; (iii) 2 h/day; (iv) 3 h/day; (v) 4 h/day; and (vi) 5 or more h/day.

The increase in screen time during the COVID-19 pandemic was assessed for each device through the question: “During the COVID-19 pandemic, has your time on television/cellphone/computer increased?” Answers were “yes” or “no.”

### Alcohol Consumption and Desire to Drink

The alcohol consumption was assessed by the question: “During the COVID-19 pandemic, how many days per week do you drink alcoholic beverages?” Answers ranged from 0 to 7 days. Those participants who reported drinking at least once a week were classified as “alcohol consumption,” for being considered as current drinkers according to Wood et al. (17).

Participants were asked about the desire to drink through the question: “During the COVID-19 pandemic, do you have an increase in the desire to drink alcoholic beverages?” Answers were “yes” or “no.” Those participants who answered “yes” for this question were classified as “increased desire to drink.”

### Smoking and Desire to Smoke

Participants' smoking habits were assessed through the question: “In the last 30 days, did you smoke?” Answers were “yes” or “no.” Those participants who answered “yes” were classified as “smokers.”

The desire to smoke was assessed by the question: “During the COVID-19 pandemic, did you have an increase in the desire to smoke?” Answers were “yes” or “no.” Those participants who answered “yes” were classified as “increased desire to smoke” even among those who said they had not smoked in the last 30 days.

### Sweetened Food Consumption

The weekly frequency of sweetened foods consumption was assessed by the question: “How many days per week do you eat sweetened foods?” Responses ranged from 0 to 7 days. Those participants who reported to eat sweetened foods for 5 or more days per week were classified as “high sweetened food consumption” (18).

Participants were also asked about how much their sweetened food consumption increased during the pandemic through the question: “During the COVID-19 pandemic, how much your sweetened food consumption increased?” Responses were: (i) nothing; (ii) increased slightly, (iii) increased moderately, and (iv) increased a lot. Participants who answered “increased moderately” and “increased a lot” were classified as “increased sweetened food consumption.”

### Covariates

Sociodemographic factors (age, sex, and educational level), mental health status (feeling of anxiety, stress, and depression), and use of screen devices for physical activity were considered as covariates. The educational level was self-reported through the question: “What is your educational level?” Answers were: (i) elementary school or less; (ii) high school; (iii) college; and (iv) post-graduate. Participants were asked about frequency that they felt stressed, anxious, and depressed during the pandemic. Responses for each feeling were: (i) never; (ii) rarely; (iii) sometimes; (iv) frequently; and (v) always. Those adults who answered “frequently” and “always” were classified for each question as having a frequent “feeling of stress,” “feeling of anxiety,” or “feeling of depression.” The use of a screen device for the practice of physical activity was assessed through the question: “During the COVID-19 pandemic, do you use social media or video conference for practicing physical activities?” Responses were “yes” or “no”.

### Statistical Analysis

Sample characteristics were presented in mean and standard deviation for continuous and in frequency for categorical variables. Binary logistic regression models were used to analyze the association between the increased screen time in each screen device and assessed outcomes: Model 1 was adjusted by sociodemographic factors (age, sex, educational level), mental health (feeling of stress, feeling of anxiety, feeling of depression), use of screen device for physical activity, and total screen time; while Model 2 was adjusted by variables from Model 1 and mutually by the other outcomes (i.e., the association between increased screen time and alcohol consumption considered smoking and sweetened food consumption as adjustments). Clusters of increased screen time, in different devices, were used to analyze whether the chance of having the outcomes was higher, according to the following categories: (i) screen time did not increase in any device (as reference); (ii) increased time in one screen device; (iii) increased time in two screen devices; and (iv) increased time in three screen devices. Analyses were performed by SPSS Statistical Package version 24.0, with significance level fixed at *p* < 0.05 and confidence interval in 95%.

## Results

A total of 1,929 adults participated in the survey, being composed by 58% of women. For this study data analysis, 33 participants were excluded due to incomplete responses, totalizing a sample of 1,896. The mean age of participants was 38.2 (13.1) years, with minimum of 18 and maximum of 88 years. The sample characteristics is presented in Table 1.

**Table 1:** Characterization of sample (*n* = 1,896).

**Categorical variables**	***n* (%)**
Sex, female	1,111 (58.6)
Educational level:	
Elementary	11 (0.6)
High school	158 (8.3)
College/graduated	807 (42.6)
Post-graduation	920 (48.5)
Feeling of stress	481 (25.4)
Feeling of anxiety	581 (30.6)
Feeling of depression	252 (13.3)
Alcohol consumption	1,245 (65.7)
Increased desire to alcohol drink during pandemic	512 (27.0)
Smoking	103 (5.4)
Increased desire to smoke during pandemic	65 (3.4)
Sweetened foods consumption for ≥5 days/week	711 (37.5)
Increased sweetened foods consumption during pandemic	807 (42.6)
Use of screen device for physical activity	709 (37.4)
Increased television time during pandemic	1,294 (68.2)
Increased cellphone time during pandemic	1,671 (88.1)
Increased computer time during pandemic	1,391 (73.4)
Cluster of screen time increased during pandemic:	
Not increased in any device	88 (4.6)
Increased in 1 device	210 (11.1)
Increased in 2 devices	635 (33.5)
Increase in the 3 devices	963 (50.8)
**Continuous variables**	**Mean (SD)**
Television time, h/day	1.7 (1.3)
Cellphone time, h/day	3.1 (1.2)
Computer time, h/day	2.5 (1.6)
Total screen time, h/day	7.2 (2.5)

*SD, standard deviation*.

The association of increased screen time in different devices with smoking, alcohol, sweetened food consumption, and desire is presented in Table 2. Adults who reported that computer time increased during the COVID-19 pandemic were less likely to report both alcohol consumption (OR = 0.68, 95% CI: 0.53; 0.86) and high sweetened food consumption (OR = 0.78, 95% CI: 0.62; 0.98). Adults whose television time increased during the COVID-19 pandemic were more likely to report increased desire to drink (OR = 1.46, 95% CI: 1.12; 1.89) and increased sweetened food consumption (OR = 1.53, 95% CI: 1.18; 1.99). Increased cell phone time was also associated with increased sweetened food consumption during the pandemic (OR = 1.78, 95% CI: 1.18; 2.67), whereas increased computer time was negatively associated with high sweetened food consumption (OR = 0.78, 95% CI: 0.62; 0.98).

**Table 2:** Association of increased time in screen devices with smoking, alcohol, sweetened food consumption, and increased desire during the COVID-19 pandemic in adults (*n* = 1,896).

	**Unadjusted**	**Adjusted** ** Model 1**	**Adjusted** ** model 2**	**Unadjusted**	**Adjusted** ** model 1**	**Adjusted** ** Model 2**
	**OR (95% CI)**	**OR (95% CI)**	**OR (95% CI)**	**OR (95% CI)**	**OR (95% CI)**	**OR (95% CI)**
	**Alcohol consumption**^**a**^ **(*****n*** **=** **1,245)**			**Increased desire to alcohol drink** ** during pandemic**^**d**^ **(*****n*** **=** **512)**		
Increased television time (*n* = 1,294)	1.07 (0.87; 1.31)	0.97 (0.78; 1.20)	0.97 (0.78; 1.21)	**1.54 (1.21; 1.96)**	**1.51 (1.16; 1.95)**	**1.46 (1.12; 1.89)**
Increased cellphone time (*n* = 1,671)	1.08 (0.80; 1.45)	1.05 (0.76; 1.43)	1.08 (0.79; 1.48)	1.34 (0.94; 1.92)	1.25 (0.86; 1.83)	1.24 (0.84; 1.82)
Increased computer time (*n* = 1,391)	0.88 (0.71; 1.10)	**0.67 (0.53; 0.86)**	**0.68 (0.53; 0.86)**	0.95 (0.75; 1.22)	0.87 (0.66; 1.14)	0.87 (0.66; 1.14)
	**Smoking**^**b**^ **(*****n*** **=** **103)**			**Increased desire to smoke during pandemic**^**e**^ **(*****n*** **=** **65)**		
Increased television time (*n* = 1,294)	0.90 (0.59; 1.36)	0.89 (0.56; 1.40)	0.87 (0.55; 1.38)	0.81 (0.48; 1.39)	0.71 (0.39; 1.30)	0.58 (0.31; 1.09)
Increased cellphone time (*n* = 1,671)	0.72 (0.41; 1.27)	0.79 (0.43; 1.45)	0.78 (0.42; 1.43)	0.84 (0.38; 1.85)	0.80 (0.34; 1.87)	0.58 (0.24; 1.40)
Increased computer time (*n* = 1,391)	0.70 (0.45; 1.07)	0.66 (0.41; 1.06)	0.71 (0.44; 1.14)	0.73 (0.41; 1.30)	0.62 (0.32; 1.18)	0.60 (0.30; 1.18)
	**Sweetened foods consumption for** **≥5 days per week**^**c**^ **(*****n*** **=** **711)**			**Increased sweetened foods consumption during pandemic**^**f**^ **(*****n*** **=** **807)**		
Increased television time (*n* = 1,294)	1.15 (0.94; 1.40)	1.02 (0.82; 1.27)	1.02 (0.83; 1.27)	**1.77 (1.45; 2.17)**	**1.56 (1.25; 1.96)**	**1.53 (1.18; 1.99)**
Increased cellphone time (*n* = 1,671)	1.16 (0.86; 1.57)	1.01 (0.73; 1.38)	1.01 (0.73; 1.38)	2.11 (1.54; 2.90)	**1.53 (1.08; 2.15)**	**1.78 (1.18; 2.67)**
Increased computer time (*n* = 1,391)	0.93 (0.75; 1.14)	**0.79 (0.63; 0.99)**	**0.78 (0.62; 0.98)**	**1.49 (1.20; 1.84)**	1.12 (0.88; 1.43)	1.16 (0.88; 1.53)

*Model 1: Adjusted by age, sex, educational level, feeling of stress, feeling of anxiety, feeling of depression, use of screen device for physical activity, and total screen time per day in each device; Model 2: Model 1 + adjusted mutually by a, b, and c, or by d, e, and f, according to the column; OR, odds ratio; CI, confidence interval. Bold values were statistically significant at p < 0.05 level*.

The Table 3 shows the association of smoking, alcohol, and sweetened food consumption and the desire with the clustering of increased time in different screen devices. Participants who reported that screen time increased in the three screen devices were less likely to have sweetened food consumption ≥5 days per week than those without an increase in any screen device during the pandemic (OR = 0.63, 95% CI: 0.39; 0.99). Otherwise, the increased time in the three screen devices was associated with twice the chance of sweetened foods consumption has been increased during pandemic (OR = 2.04, 95% CI: 1.07; 3.88).

**Table 3:** Association of the clustering of increased time in different devices with smoking, alcohol, sweetened food consumption, and increased desire during the COVID-19 pandemic in adults (*n* = 1,896).

	**Unadjusted**	**Adjusted** ** model 1**	**Adjusted** ** model 2**	**Unadjusted**	**Adjusted** ** model 1**	**Adjusted** ** model 2**
	**OR (95% CI)**	**OR (95% CI)**	**OR (95% CI)**	**OR (95% CI)**	**OR (95% CI)**	**OR (95% CI)**
	**Alcohol consumption**^**a**^ **(*****n*** **=** **1,245)**			**Increased desire to alcohol drink during pandemic**^**d**^ **(*****n*** **=** **512)**		
Screen time did not increase (*n* = 88)	1.00 (Reference)	1.00 (Reference)	1.00 (Reference)	1.00 (Reference)	1.00 (Reference)	1.00 (Reference)
Increased time in 1 screen device (*n* = 210)	1.04 (0.62; 1.73)	1.01 (0.59; 1.73)	1.06 (0.62; 1.81)	1.24 (0.63; 2.44)	1.20 (0.61; 2.34)	1.21 (0.62; 2.38)
Increased time in 2 screen devices (*n* = 635)	1.28 (0.80; 2.03)	1.16 (0.71; 1.88)	1.23 (0.75; 2.00)	1.72 (0.93; 3.17)	1.68 (0.92; 3.07)	1.63 (0.89; 3.01)
Increased time in 3 screen devices (*n* = 963)	1.11 (0.71; 1.74)	0.85 (0.52; 1.38)	0.90 (0.55; 1.46)	1.79 (0.98; 3.27)	1.62 (0.89; 2.97)	1.56 (0.84; 2.87)
	**Smoking**^**b**^ **(*****n*** **=** **103)**			**Increased desire to smoke during pandemic**^**e**^ **(*****n*** **=** **65)**		
Screen time did not increase (*n* = 88)	1.00 (Reference)	1.00 (Reference)	1.00 (Reference)	1.00 (Reference)	1.00 (Reference)	1.00 (Reference)
Increased time in 1 screen device (*n* = 210)	0.88 (0.35; 2.25)	0.86 (0.33; 2.23)	0.86 (0.33; 2.24)	0.56 (0.15; 2.14)	0.81 (0.21; 3.16)	0.68 (0.17; 2.69)
Increased time in 2 screen devices (*n* = 635)	0.59 (0.25; 1.38)	0.60 (0.25; 1.43)	0.58 (0.24; 1.39)	0.58 (0.18; 1.88)	0.93 (0.28; 3.13)	0.65 (0.19; 2.24)
Increased time in 3 screen devices (*n* = 963)	0.63 (0.28; 1.43)	0.61 (0.26; 1.44)	0.61 (0.26; 1.47)	0.50 (0.16; 1.56)	0.66 (0.20; 2.20)	0.45 (0.13; 1.55)
	**Sweetened foods consumption for** **≥5 days per week**^**c**^ **(*****n*** **=** **711)**			**Increased sweetened foods consumption during pandemic**^**f**^ **(*****n*** **=** **807)**		
Screen time did not increase (*n* = 88)	1.00 (Reference)	1.00 (Reference)	1.00 (Reference)	1.00 (Reference)	1.00 (Reference)	1.00 (Reference)
Increased time in 1 screen device (*n* = 210)	0.66 (0.39; 1.10)	0.60 (0.36; 1.02)	0.60 (0.35; 1.02)	1.34 (0.74; 2.43)	1.00 (0.56; 1.79)	1.08 (0.53; 2.21)
Increased time in 2 screen devices (*n* = 635)	0.83 (0.53; 1.31)	0.68 (0.43; 1.09)	0.68 (0.43; 1.09)	**2.10 (1.23; 3.60)**	1.27 (0.75; 2.16)	1.59 (0.84; 3.04)
Increased time in 3 screen devices (*n* = 963)	0.83 (0.53; 1.30)	**0.62 (0.39; 0.99)**	**0.63 (0.39; 0.99)**	**3.13 (1.85; 5.32)**	**1.74 (1.03; 2.94)**	**2.04 (1.07; 3.88)**

*Model 1: Adjusted by sex, age, educational level, use of screen device for physical activity, and total time spent in screen time per day; Model 2: Model 1 + adjusted mutually by a, b, and c, or by d, e, and f, according to the column; OR, odds ratio; CI, confidence interval. Bold values were statistically significant at p < 0.05 level*.

## Discussion

This study observed that increased time in television and cell phone usage was associated with addictive behaviors and increased substance craving during the COVID-19 pandemic, while increased time on the computer was negatively associated with consumption of alcohol and sweetened foods. Adults with increased time in the three devices (television, cell phone, and computer) were less likely to consume sweetened foods for ≥5 days per week but were more likely to have their sweetened food consumption increased during the pandemic.

Increased time watching television was associated with an increased desire for alcohol consumption in this study. This result may be related to a larger exposure to advertisements on television, since the association of advertisements with alcohol consumption and alcohol-related cognitions has been described even before the COVID-19 pandemic (19), and greater alcohol craving was an observed event in the home environment (20). Although the moderate alcohol consumption has been associated with psychological benefits, such as stress reduction, happiness, and decreases in tension and depression since decades prior (21), frequent alcohol consumption results in neuroadaptive changes (22), which is associated with alcohol dependence (23). Therefore, the increases in screen time and increased desire to alcohol drink during the COVID-19 pandemic can be a potent trigger to higher alcohol consumption and consequently alcohol dependence in the future. In addition, both excessive alcohol consumption and high television viewing have been associated with psychological distress and moderate-to-severe depression in adults (24, 25), which may also be aggravated by such difficult times due to the pandemic.

Television viewing was also associated with increased sweetened food consumption in the present study. The consumption of sweetened foods and other energy-dense snacks have been positively associated with television viewing in adults even before the pandemic (26, 27). The link between television and sweetened food consumption can be related with the mentally passive characteristics of television viewing that allows concomitant eating behaviors and could also increase the risk of depression (28). In addition, television has also presenting excessive content about the COVID-19 pandemic, which may cause negative effects in mood and boredom, and sweetened food consumption could counteract the frequent news about the current period by the releasing of dopamine, which activates pleasurable and rewarding sensations, improving psychological well-being (29).

This study also observed that increased time on the computer was a protective factor for both alcohol and high-sweetened food consumption during the pandemic. A possible hypothesis is that computer time could be mostly related to occupational tasks, such as home-office and virtual classes, which leads to a higher time spent in mentally active screen behaviors. It has been previously observed that mentally active screen behaviors as computer use have been related with better mental health (30) and higher moderate-to-vigorous physical activity (31), which presented a protective role in the physical and nutritional health impairments of the pandemic (32).

The cluster of increased screen time was associated with increased sweetened food consumption during the pandemic. It is possible that increased time in different devices may be related to higher exposure to unhealthy food advertisements with negative effects on food choice, since previous pandemic studies reported that people who were exposed to food advertising chose 28% more unhealthy snacks when compared to those who were exposed to non-food advertisements (6). However, the present study also observed that adults with increased time in three screen devices were less likely to have sweetened food consumption for 5 days per week and more, even being more likely to have their consumption increased during the pandemic. It is possible that increased sweetened food consumption during the pandemic was not sufficient to make it more frequent, although it was not known whether the portions per day could have been increased.

Although we have filtered discrepant and improbable responses to improve the data quality, this study was susceptible to information bias, as well as the invitation procedures precluded participation of individuals without access to social media. The total weekly amount of drinking was not assessed, as well as the daily quantity of sweetened foods consumed, which would add valuable information. This study did not consider which body position the screen devices were used, which compromise inferences about sedentary behavioral patterns related to screen time. The lack of information about the employment status and labor activities may be seen as a limitation of the study, as well as other reasons for increases in screen time during the pandemic. The adjustments for mental health, sociodemographic factors, and total time spent on screen devices in the analysis by different screen devices were the strengths of the study.

## Conclusion

During the COVID-19 pandemic, increased screen time was differently associated with alcohol and sweets according to screen devices. Increased television and cell phone time was associated with increased sweetened food consumption and increased desire to drink alcoholic beverages, while increased computer time was a protective factor for both alcohol and high sweetened food consumption. The increased screen time spent on the television and cell phone needs to be considered for further investigation of unhealthy behaviors caused by the pandemic.

## Data Availability Statement

The raw data supporting the conclusions of this article will be made available by the authors, without undue reservation.

## Ethics Statement

The studies involving human participants were reviewed and approved by Universidade Nove de Julho' Ethics Committee before data collection (CAAE #30890220.4.0000.5511). The patients/participants provided their written informed consent to participate in this study.

## Author Contributions

WRT, DGDC, and TAD: conceptualization, data analysis, writing, and manuscript draft. MCL-P, JPB, MAC, and GGC: writing and data curation. WLP and RMR-D: conceptualization and writing. All the authors approved the final version of manuscript.

## Conflict of Interest

The authors declare that the research was conducted in the absence of any commercial or financial relationships that could be construed as a potential conflict of interest.
